# Biochemical characterization of the α-1,3-mannosidase AnGH92A from *Aspergillus nidulans*

**DOI:** 10.1038/s41598-025-34466-6

**Published:** 2026-01-05

**Authors:** Ryutaro Nishigaki, Hiromitsu Suzuki, Ryohei Tsukada, Ken Miyazawa, Masashi Kato, Motoyuki Shimizu

**Affiliations:** 1 Faculty of Agriculture, Meijo University, Nagoya, Japan; 2 Department of Fungal Infection, National Institute of Infectious Diseases, Japan Institute for Health Security, Tokyo, Japan

**Keywords:** α-Manno-oligosaccharides, α-1,3-Mannosidase, Glycoside hydrolases, GH92 family, *Aspergillus nidulans*, Biochemistry, Biotechnology, Microbiology, Structural biology

## Abstract

α-Mannan is a structurally diverse polysaccharide widely distributed in fungi, yet the eukaryotic enzymes responsible for its degradation remain poorly understood. Glycoside hydrolase family 92 (GH92) enzymes, well characterized in bacteria as Ca^2^⁺-dependent *exo*-α-mannosidases, have largely uncharacterized biochemical roles in fungi. Here, we characterized two GH92 enzymes from the filamentous fungus *Aspergillus nidulans*, designated AnGH92A and AnGH92B. Both recombinant enzymes hydrolyzed 4-nitrophenyl α-d-mannopyranoside (4NP-Man), but only AnGH92A displayed activity toward natural substrates. AnGH92A exhibited high specificity for α-1,3-linked mannooligosaccharides, weak activity toward α-1,4-linkages, and no detectable activity toward α-1,2- or α-1,6-linkages. It also cleaved α-1,3-linked side chains in branched oligosaccharides and released mannose from yeast α-mannan. By contrast, recombinant AnGH92B showed no detectable hydrolytic activity toward α-manno-oligosaccharides and yeast α-mannan. Structural modeling revealed pronounced differences in the substrate-binding pockets of AnGH92A and AnGH92B. Site-directed mutagenesis confirmed that Glu598 and Asp685 serve as the general acid and general base catalysts of AnGH92A, respectively, and that Tyr588 is essential for substrate recognition. Together, these findings indicate that AnGH92A functions as a eukaryotic GH92 α-1,3-mannosidase with weak activity toward α-1,4-linkages and provide the first experimental evidence linking sequence motifs and active-site architecture to catalytic function in fungal GH92 enzymes.

**Supplementary Information:**

The online version contains supplementary material available at 10.1038/s41598-025-34466-6.

## Introduction

α-Mannan is a structurally diverse polysaccharide widely distributed in fungi, localized both as a component of the cell wall and as *N*- and *O*-linked glycans of glycoproteins^1–3^. In the yeast *Saccharomyces cerevisiae*, the *N*-glycan outer chain consists of an α-1,6-linked mannose backbone with α-1,2- and α-1,3-linked side chains, forming a highly branched structure of more than 200 residues^4–7^. In filamentous fungi, α-mannan also occurs in cell wall polysaccharides such as fungal-type galactomannan (FTGM) and *O*-mannose-type galactomannan (OMGM)^8–15^. Beyond their structural roles, these glycans function as antigens recognized by the host immune system^16–20^.

The degradation of α-mannan involves six glycoside hydrolase (GH) families—GH38, GH47, GH76, GH92, GH99, and GH125^21^. In bacteria, enzymes from these families act in a coordinated manner, with distinct α-mannosidases targeting specific glycosidic linkages and structural motifs. This coordination enables the stepwise degradation of the highly branched α-mannan structure^22–24^. Notably, putative α-mannosidases belonging to GH76, GH92, and GH125 are found exclusively in fungi and are absent in animals and plants^21^, suggesting that fungi have evolved a specialized repertoire of glycoside hydrolases for α-mannan turnover within their own cell walls or from environmental sources. Among these, the *dfg5* and *dcw1* genes encoding GH76 enzymes in *Neurospora crassa* function as α-1,6-mannanases that cleave *N*-linked galactomannan, thereby crosslinking glycoproteins to the glucan–chitin matrix and contributing to cell wall assembly^25^. Similar roles have been reported in *Candida albicans*, where GH76 enzymes are required for cell wall integrity and morphogenesis^26–29^. In contrast, although GH92 and GH125 enzymes from prokaryotes have been extensively characterized, their functional roles in eukaryotes remain unclear.

Bacterial GH92 enzymes are predominantly Ca^2^⁺-dependent *exo-*α-mannosidases that hydrolyze α-1,2-, α-1,3-, and α-1,4-linked mannose residues in α-mannan^30–32^. They play central roles in the degradation of yeast α-mannans and high-mannose–type *N*-glycans in fungal and plant glycoproteins^22,33–36^. Extensive biochemical and structural analyses of bacterial GH92 enzymes, particularly from gut microbes, have revealed diverse substrate specificities and catalytic mechanisms^22,30–38^. However, GH92 enzymes in eukaryotes, especially fungi, remain poorly understood, and their substrate preferences and physiological functions are largely unexplored.

To investigate the catalytic diversity of eukaryotic GH92 enzymes, we focused on those encoded in the genome of the filamentous fungus *Aspergillus nidulans*, a classical model organism widely used in genetics and molecular biology. The *A. nidulans* genome encodes five putative GH92 genes: *ANIA_10672*, *ANIA_01197*, *ANIA_03054*, *ANIA_02325*, and *ANIA_03764* which encode the proteins designated as AnGH92A, AnGH92B, AnGH92C, AnGH92D, and AnGH92E, respectively. Among them, AnGH92A, AnGH92B, and AnGH92C possess predicted N-terminal signal peptides, suggesting that they function as secreted enzymes. In this study, we cloned three GH92 genes *ANIA_10672* (AnGH92A), *ANIA_01197* (AnGH92B), and *ANIA_03054* (AnGH92C); however, AnGH92C could not be produced using the heterologous expression system, and substrate specificity could not be determined for AnGH92B due to its limited activity. Therefore, we focused primarily on AnGH92A to elucidate its substrate specificity, predicted three-dimensional structure, and catalytic mechanism.

## Materials and methods

### Chemicals and reagents

α-1,2-Mannobiose (α-1,2-Man_2_), α-1,3-mannobiose (α-1,3-Man_2_), α-1,4-mannobiose (α-1,4-Man_2_), α-1,6-mannobiose (α-1,6-Man_2_), *O*-α-d-mannopyranosyl-(1→3)-*O*-[α-d-mannopyranosyl-(1→6)]-α-d-mannopyranose (Man_3_), *O*-α-d-mannopyranosyl-(1→3)-*O*-[α-d-mannopyranosyl-(1→6)]-*O*-α-d-mannopyranosyl-(1→6)-d-mannose (Man_4_), and *O*-α-d-mannopyranosyl-(1→3)-*O*-[α-d-mannopyranosyl-(1→6)]-*O*-α-d-mannopyranosyl-(1→6)-*O*-[α-d-mannopyranosyl-(1→3)]-d-mannose (Man_5_) were purchased from Dextra Laboratories (Reading, UK). 4-Nitrophenyl α-d-mannopyranoside (4NP-Man) and *S. cerevisiae* mannan were obtained from Sigma-Aldrich (St. Louis, MO, USA).

### Strains, culture, and media

The *A. nidulans* A26 strain, obtained from the Fungal Genetic Stock Center (Kansas State University, Manhattan, KS, USA), was maintained at 37 °C on minimal medium (MM) agar containing 10 mM NaNO₃, 10 mM KH₂PO₄, 7 mM KCl, 2 mM MgSO₄, 2 mL/L Hutner’s trace metals, and 1.5% (w/v) agar (initial pH 6.5), supplemented with 1.0% (w/v) glucose as the sole carbon source. For liquid culture, conidia (2.0 × 10⁶) were inoculated into 100-mL Erlenmeyer flasks containing 20 mL MM with 1.0% (w/v) glucose and incubated at 37 °C on a rotary shaker at 100 rpm^39^.

### Construction of a phylogenetic tree

For phylogenetic analysis of GH92 proteins, amino acid sequences of 32 functionally characterized bacterial GH92 enzymes were retrieved from the Carbohydrate-Active enZymes (CAZy) database (http://www.cazy.org/)^21^, along with representative fungal GH92 sequences (e.g., *Aspergillus* and *Candida* spp.). Multiple sequence alignment was performed using MUSCLE (MUltiple Sequence Comparison by Log-Expectation) implemented in *Geneious Prime* (Biomatters Ltd., Auckland, New Zealand). Phylogenetic trees were constructed using the neighbor-joining method, and branch robustness was assessed by 1,000-replicate bootstrap analysis.

### Cloning of AnGH92A and AnGH92B genes from *A. nidulans*

Full-length GH92 genes from *A.nidulans* were amplified by polymerase chain reaction (PCR) using the primer sets listed in Table S1 and a DNA Thermal Cycler 2400 (Takara Bio, Otsu, Japan). PCR amplification consisted of 30 cycles of denaturation at 98 °C for 10 s, annealing at 65 °C for 5 s, and extension at 68 °C for 5 s. Amplified products were separated on 1% (w/v) agarose gels, stained with ethidium bromide, and visualized using a Molecular Imager FX (Bio-Rad, Hercules, CA, USA). PCR products were ligated into the pPICZα-A expression vector (Novagen, Darmstadt, Germany), and the resulting plasmids were introduced into *Escherichia coli* JM109 (Invitrogen, Carlsbad, CA, USA) by heat-shock transformation. Transformants were selected on Luria–Bertani (LB) agar plates containing Zeocin (25 µg/mL), and positive clones were verified by DNA sequencing. Site-directed mutagenesis was performed by inverse PCR using mutagenic primers listed in Table S1. Following amplification, parental plasmid DNA was digested with *Dpn*I, and mutated plasmids were transformed into *E. coli* JM109. Plasmid DNA isolated from selected colonies was sequenced for confirm the presence of the intended mutations.

### Preparation of recombinant AnGH92 enzymes

The recombinant pPICZα-A plasmids carrying the AnGH92 genes were transformed into *Komagataella phaffii* (formerly *Pichia pastoris*) KM71H (Invitrogen) for heterologous protein expression, and recombinant strains were cultured as previously described^40^. After induction, culture supernatants were harvested by centrifugation and concentrated using an Amicon Ultra centrifugal filter unit equipped with a regenerated cellulose membrane (molecular weight cutoff 30,000; Merck Millipore, Billerica, MA, USA). Concentrated samples were applied to a HiTrap DEAE FF anion-exchange column (GE Healthcare), and bound proteins were eluted with 20 mM Tris-HCl (pH 8.0) containing 100 mM NaCl. Fractions containing recombinant protein were further purified by size-exclusion chromatography on a Superose 6 10/300 GL column (GE Healthcare) equilibrated with 20 mM Tris-HCl (pH 8.0) and 150 mM NaCl. Protein-containing fractions were pooled and dialyzed against 20 mM Tris-HCl (pH 8.0). All purification steps were performed at 4 °C to maintain protein stability. Protein concentrations were determined using the Bradford assay.

### Enzyme assays

α-Mannosidase activity was measured using 4-nitrophenyl-α-d-mannopyranoside (4NP-Man) as the substrate^30^. Each reaction mixture (200 µL total volume) contained 2.0 mM CaCl₂, 50 mM sodium acetate buffer (pH 5.0), 1.0 mM 4NP-Man, and purified enzyme. Reactions were initiated by enzyme addition and incubation at 40 °C. The release of 4-nitrophenol (4NP) was monitored spectrophotometrically at 405 nm using a microplate reader (Molecular Devices, San Jose, CA, USA). All assays were performed in triplicate using independently prepared enzyme samples.

Initial reaction rates were calculated from the amount of 4NP released. The apparent kinetic parameters, Michaelis constant (*K*_m_) and turnover number (*k*_cat_), were determined by fitting initial rates to the Michaelis–Menten equation using nonlinear regression in Origin version 6.0 (OriginLab, Northampton, MA, USA). Reaction products generated from α-manno-oligosaccharides and α-mannan were analyzed by thin-layer chromatography (TLC) on Silica gel 60 plates (Merck Millipore) developed with *n*-butanol:acetic acid:water (9:4:7, v/v/v). Dried plates were sprayed with ethanol containing 0.82% (w/v) N-(1-naphthyl)ethylenediamine dihydrochloride and 5.0% (v/v) sulfuric acid (98%), and heated at 105 °C for 3 min to visualize reaction products.

Soluble hydrolysis products were additionally analyzed by high-performance liquid chromatography (HPLC) using a Prominence reducing-sugar analytical system (Shimadzu, Kyoto, Japan) equipped with a fluorescence detector. Separation was performed on a Shim-pack ISA-07/S2504 column (4.0 × 250 mm; Shimadzu) using a linear gradient from 0.1 M potassium borate buffer (pH 8.0) to 0.4 M potassium borate buffer (pH 9.0) over 90 min at a flow rate of 0.6 mL/min. Quantification was performed using mannose, α-1,2-Man_2_, α-1,3-Man_2_, α-1,4-Man_2_, α-1,6-Man_2_, Man_3_, Man_4_, and Man_5_ as standards. Hydrolytic reaction rates toward α-manno-oligosaccharides and α-mannan were determined by monitoring the mannose release by HPLC. Initial rates toward α-1,3-Man_2_ were calculated from the amount of mannose produced, and kinetic parameters (*k*_cat_ and *K*_m_) were determined using the same nonlinear regression approach described above.

### Determination of optimal pH and temperature

The optimal pH for enzyme activity was evaluated across pH 3.0–10.0 using the following buffer systems: 50 mM sodium acetate (pH 3.0–6.0), 50 mM sodium phosphate (pH 6.0–8.0), and 50 mM Tris-HCl (pH 8.0–10.0). Reactions were performed at 40 °C for 60 min, and enzymatic activity was measured using 4NP-Man as the substrate. The optimal temperature was determined by incubating the enzyme with 4NP-Man in 50 mM sodium acetate buffer (pH 5.0) at temperatures ranging from 20 °C to 90 °C for 60 min. All data are presented as mean ± standard deviation from three independent experiments.

### Effect of metal ions

To assess the influence of metal ions on enzyme activity, purified enzyme (1.0 µM final concentration) was incubated for 24 h at 4 °C in 50 mM sodium acetate buffer (pH 5.0) containing 2.0 mM CaCl₂, CuCl₂, ZnCl₂, CoCl₂, MgCl₂, or the chelating agent ethylenediaminetetraacetic acid (EDTA). After incubation, residual activity was measured under standard assay conditions (pH 5.0, 37 °C, 30 min) using 1.0 mM 4NP-Man as the substrate.

### Structural analysis of AnGH92 enzymes

Structural models of AnGH92 enzymes were generated using AlphaFold2^41^. Docking simulations were carried out the Molecular Operating Environment (MOE; Chemical Computing Group, Montreal, QC, Canada). The ligand α-1,3-Man₂ (PubChem CID: 11013287), which adopts a ^4^*C*₁ chair conformation in its lowest-energy state, was retrieved from the PubChem database. To reproduce the conserved catalytic geometry of bacterial GH92 enzymes, a Ca^2^⁺ ion was manually placed into the modelled structure of AnGH92A based on the Ca^2^⁺-binding coordinates of the BT3990 crystal structure (PDB ID: 2WW1), and docking simulations were performed using this Ca^2^⁺-bound model. Docking poses and active-site interactions were visualized and analyzed using PyMOL version 2.5.

## Results

### Phylogenetic clustering of fungal GH92 enzymes

**Fig. 1:**
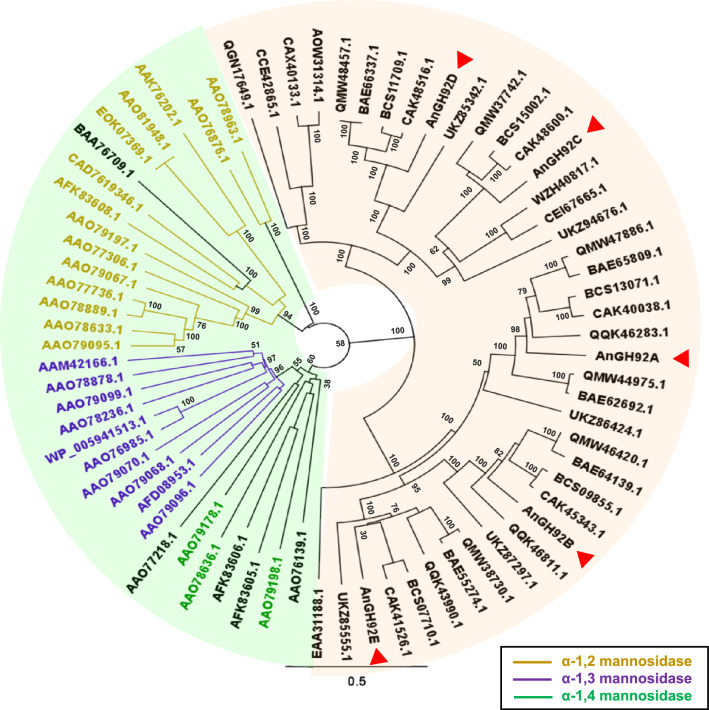
Phylogenetic analysis of glycoside hydrolase family 92 (GH92) enzymes. The phylogenetic tree was generated using Geneious Prime with the neighbor-joining method and 1,000 bootstrap replicates. Protein sequences were retrieved from the UniProt, GenBank, and CAZy databases. Background shading indicates taxonomic origin: bacterial sequences are shown in green; *Aspergillus* and *Candida* species sequences are shown in beige. Branch colors denote enzyme source or substrate specificity: yellow, α-1,2-mannosidases; purple, α-1,3-mannosidases; green, α-1,4-mannosidases. Arrowheads indicate GH92 enzymes AnGH92A (*ANIA_10672*), AnGH92B (*ANIA_01197*), AnGH92C (*ANIA_03054*), AnGH92D (*ANIA_02325*), and AnGH92E (*ANIA_03764*) from *Aspergillus nidulans*.

Five GH92 genes—*ANIA_10672*, *ANIA_01197*, *ANIA_03054*, *ANIA_02325*, and *ANIA_03764*—are present in the *A. nidulans* genome, encoding the proteins, AnGH92A, AnGH92B, AnGH92C, AnGH92D, and AnGH92E, respectively (Fig. 1; Table S2). Among these proteins, AnGH92A, AnGH92B, and AnGH92C contain predicted N-terminal signal peptides, suggesting that they are secreted enzymes. A phylogenetic tree was constructed using 32 biochemically characterized bacterial GH92 enzymes and 41 fungal GH92 representatives, together with the five *A. nidulans* sequences (Fig. 1). Bacterial GH92 enzymes grouped into three well-resolved clades corresponding to α-1,2-, α-1,3-, and α-1,4-mannosidase activities, consistent with established relationships between sequence homology and substrate specificity. In contrast, fungal GH92 enzymes clustered separately from bacterial sequences. Within fungi, AnGH92A, AnGH92B, and AnGH92E formed one clade, whereas AnGH92C and AnGH92D each occupied distinct clades. GH92 enzymes occur exclusively in fungi among eukaryotes, with no homologs identified in animals or plants, suggesting that GH92 genes have been uniquely retained and diversified during fungal evolution. To investigate the biochemical properties of these enzymes, we attempted heterologous expression of the three predicted secreted proteins—AnGH92A, AnGH92B, and AnGH92C—from *A. nidulans*.

### Catalytic properties of AnGH92 enzymes

**Fig. 2:**
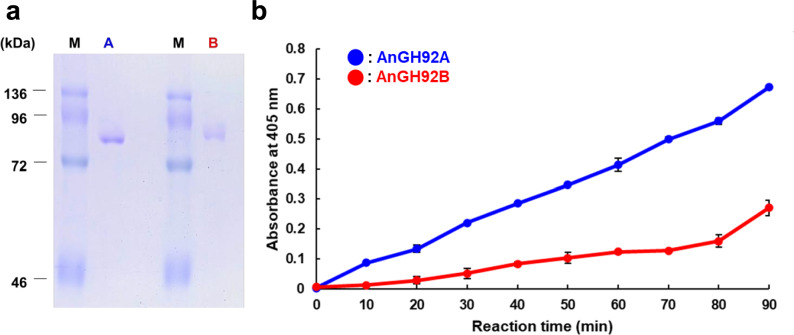
Recombinant production and activity assays of AnGH92A and AnGH92B. (**a**) SDS-PAGE analysis of recombinant AnGH92A and AnGH92B. Lane A, AnGH92A; Lane B, AnGH92B; Lane M, protein molecular mass markers. (**b**) α-Mannosidase activity using 4NP-Man as the substrate. The release of 4NP was monitored at 405 nm. Blue circles, AnGH92A; red circles, AnGH92B. Values represent the mean ± standard error from three independent experiments.

To assess their catalytic activities, the three GH92 proteins from *A. nidulans* were heterologously expressed in *K. phaffii*. Purified AnGH92A and AnGH92B migrated as single bands on sodium dodecyl sulfate–polyacrylamide gel electrophoresis (SDS–PAGE) (Fig. 2a). The observed molecular masses (87 kDa and 88 kDa, respectively) were consistent with values predicted from their amino acid sequences. By contrast, AnGH92C could not be successfully produced in the *K. phaffii* expression system (data not shown).

The hydrolytic activities of AnGH92A and AnGH92B, which share 46.2% amino acid sequence identity, were first examined using the model substrate 4NP-Man. The release of 4NP, which absorbs maximally at 405 nm under the assay conditions, was monitored spectrophotometrically. Both enzymes exhibited time-dependent increases in absorbance, confirming α-mannosidase activity (Fig. 2b). Kinetic parameters for AnGH92A and AnGH92B were determined using 4NP-Man, revealing that although AnGH92B displayed a higher *K*_m_ value, the catalytic efficiencies (*k*_cat_/*K*_m_) of the two enzymes were comparable (Table 1).

**Table 1:** Kinetic parameters of AnGH92A and AnGH92B toward 4NP-Man and α-1,3-Man_2_.

**Enzyme**	**4NP-Man**			**α-1,3-Man₂**		
	*k*_cat_ (min^-1^)	*K*_m_ (mM)	*k*_cat_/*K*_m_ (min^-1^mM^-1^)	*k*_cat_ (min^-1^)	*K*_m_ (mM)	*k*_cat_/*K*_m_ (min^-1^mM^-1^)
AnGH92A	114 ± 1.6	2.4 ± 0.36	49	3.5 × 10^4^ ± 1.2 × 10^3^	20 ± 2.8	1.8×10^3^
AnGH92B	552 ± 2.4	20.1 ± 0.079	27	-	-	-

The apparent kinetic parameters were determined by nonlinear regression fitting of initial reaction rates to the Michaelis–Menten equation. Values represent fitted parameters ± standard error form three independent experiments.

The effects of pH, temperature, and metal ions on enzyme activity were also evaluated. Both AnGH92A and AnGH92B displayed maximal activity at 40 °C and pH 5.0 (Supplementary Fig. S1). Enzyme activity increased in the presence of Ca^2^⁺, whereas treatment with EDTA markedly reduced activity, indicating that metal ions are required for catalysis (Supplementary Figs. S1c, S1d). This metal dependence is consistent with previous reports on bacterial GH92 enzymes^30,31,36,42^.

### Substrate specificity of AnGH92 enzymes

**Fig. 3:**
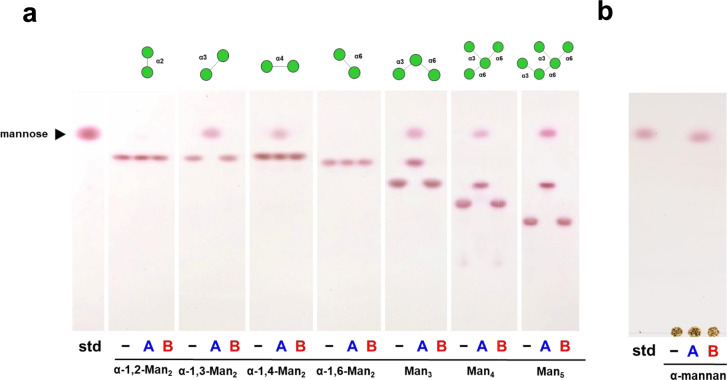
TLC analysis of reaction products generated by AnGH92A and AnGH92B. (**a**, **b**) Reaction products from α-manno-oligosaccharides (**a**) and α-mannan (**b**). Reactions were carried out at 40 °C and pH 5.0 for 24 h. Std, mannose standard; −, reaction without enzyme; A, reaction with AnGH92A; B, reaction with AnGH92B.

**Fig. 4:**
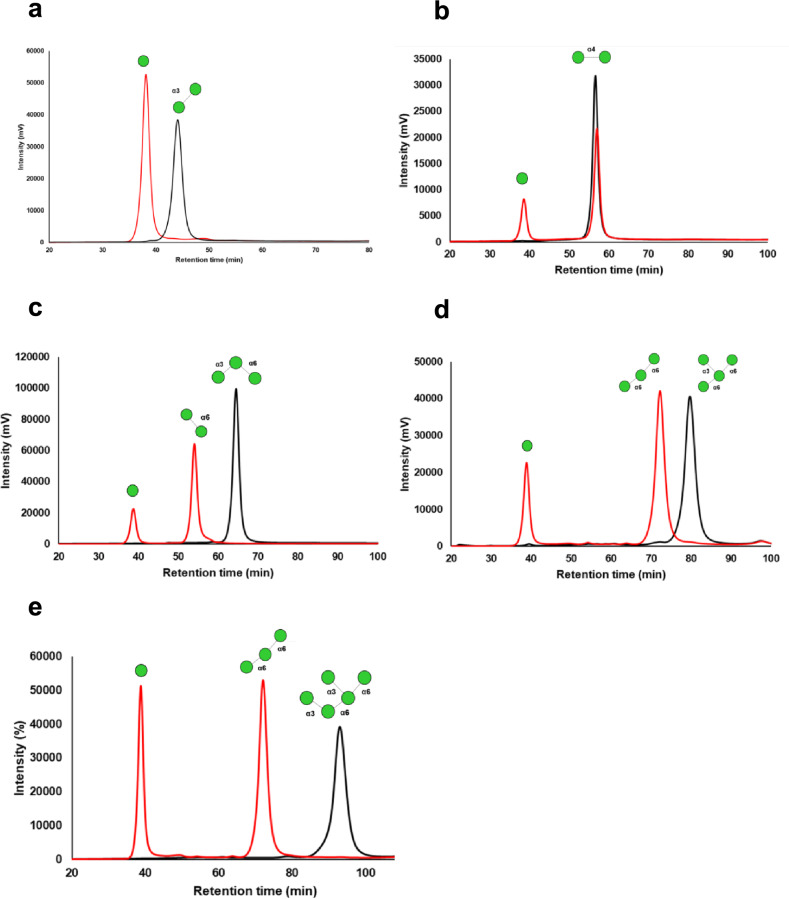
HPLC analysis of hydrolysis products generated from α-manno-oligosaccharides by AnGH92A. Chromatograms at 0 min (black) and 30 min (red) are shown for reactions with α-manno-oligosaccharide substrates hydrolyzed by AnGH92A. (a–e) Reaction products were analyzed using a reducing-sugar HPLC system, and peaks were identified based on retention times of authentic standards. Substrate peaks decreased or disappeared after 30 min of incubation, while new peaks corresponding to hydrolysis products appeared. Each experiment was performed in triplicate, and representative chromatograms are shown. Substrates were: (**a**) α-1,3-mannobiose; (**b**) α-1,4-mannobiose; (**c**) *O*-α-d-mannopyranosyl-(1→3)-*O*-[α-d-mannopyranosyl-(1→6)]-α-d-mannopyranose (Man₃); (**d**) *O*-α-d-mannopyranosyl-(1→3)-*O*-[α-d-mannopyranosyl-(1→6)]-*O*-α-d-mannopyranosyl-(1→6)-d-mannose (Man₄); (**e**) *O*-α-d-mannopyranosyl-(1→3)-*O*-[α-d-mannopyranosyl-(1→6)]-*O*-α-d-mannopyranosyl-(1→6)-*O*-[α-d-mannopyranosyl-(1→3)]-d-mannose (Man₅).

**Fig. 5:**
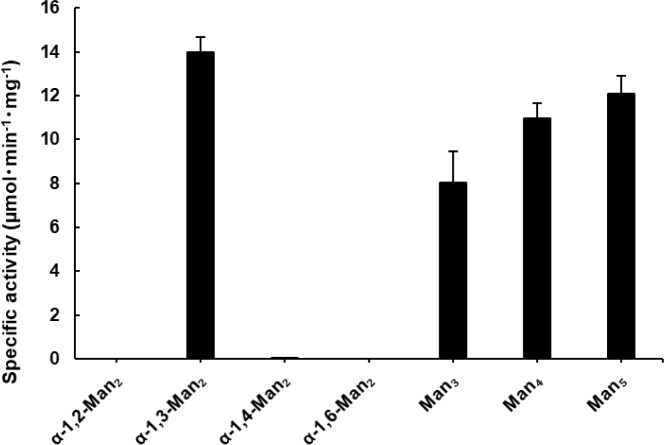
Substrate specificity of AnGH92A. Specific activities (µmol·min⁻^1^·mg⁻^1^) were determined for α-manno-oligosaccharides: α-1,2-mannobiose (α-1,2-Man₂); α-1,3-mannobiose (α-1,3-Man₂); α-1,4-mannobiose (α-1,4-Man₂); α-1,6-mannobiose (α-1,6-Man₂); *O*-α-d-mannopyranosyl-(1→3)-*O*-[α-d-mannopyranosyl-(1→6)]-α-d-mannopyranose (Man₃); *O*-α-d-mannopyranosyl-(1→3)-*O*-[α-d-mannopyranosyl-(1→6)]-*O*-α-d-mannopyranosyl-(1→6)-d-mannose (Man₄); and *O*-α-d-mannopyranosyl-(1→3)-*O*-[α-d-mannopyranosyl-(1→6)]-*O*-α-d-mannopyranosyl-(1→6)-*O*-[α-d-mannopyranosyl-(1→3)]-d-mannose (Man₅). Specific activities were calculated from mannose release quantified by HPLC. Values represent the mean ± standard error from three independent experiments.

The substrate specificities of recombinant AnGH92 enzymes were examined using a panel of α-manno-oligosaccharides and α-mannan, with reaction products analyzed by TLC and HPLC. The substrates included four α-mannobioses (α-1,2-Man_2_, α-1,3-Man_2_, α-1,4-Man_2_, and α-1,6-Man_2_), branched α-manno-oligosaccharides containing α-1,3- and α-1,6-linkages (Man₃, Man₄, and Man₅), and *S. cerevisiae* cell-wall α-mannan (Fig. 3, 4). AnGH92A hydrolyzed a subset of these substrates, whereas no detectable activity was observed for AnGH92B (Fig. 3a, b). As shown in Fig. 5, AnGH92A exhibited the highest specific activity toward α-1,3-Man₂, reduced activity toward α-1,4-Man₂, and no activity toward α-1,2-Man_2_ or α-1,6-Man₂. These findings demonstrate that AnGH92A possesses high substrate specificity, efficiently cleaving α-1,3-linkages while displaying only weak activity toward α-1,4-linkages. Consistent with this specificity, AnGH92A completely hydrolyzed the α-1,3-linked side chains of branched oligosaccharides (Man₃, Man₄, and Man₅) (Fig. 3a, 4). TLC and HPLC analyses showed that hydrolysis of Man₃ yielded mannose and α-1,6-Man₂, whereas Man₄ and Man₅ produced mannose and an α-1,6-linked mannotriose (Fig. 4). Kinetic parameters revealed that the catalytic efficiency of AnGH92A toward α-1,3-Man₂ was approximately 36-fold higher than that measured using 4NP-Man (Table 1).

AnGH92A also exhibited hydrolytic activity toward yeast α-mannan, releasing mannose as the major product. Because *S. cerevisiae* cell-wall mannan consists of an α-1,6-linked backbone with numerous α-1,2- and α-1,3-linked side chains, mannose release is most likely due to cleavage of the α-1,3-linked substituents (Fig. 3b). As summarized in Fig. 5, α-1,3-Man₂ was the most efficiently hydrolyzed substrate, followed by the branched oligosaccharides Man_5_, Man₄ and Man₃. Activity toward the α-1,4-linked disaccharide was substantially lower, and no hydrolysis was detected for α-1,2- or α-1,6-linked substrates. Collectively, these results establish AnGH92A as an α-1,3-mannosidase with weak activity toward α-1,4-linkages.

### Comparison of predicted structures of AnGH92A and AnGH92B

Structural models of AnGH92A and AnGH92B were generated using AlphaFold2 (Supplementary Fig. S2). Both enzymes displayed the characteristic two-domain architecture of GH92 α-mannosidases, consisting of an N-terminal β-sandwich domain and a C-terminal (α/α)₆ barrel fold (Supplementary Fig. S2). The active sites were positioned in shallow grooves at the interface of the two domains, with residues from both regions contributing to pocket formation, consistent with reported GH92 crystal structures^30–33,36,42^.

GH92 enzymes are Ca^2^⁺-dependent, and conserved Ca^2^⁺-binding residues were identified within the predicted active sites of AnGH92A and AnGH92B (Supplementary Fig. S3). Structural modeling supports a role for Ca^2^⁺ in stabilizing substrate binding and facilitating catalysis. Accordingly, Ca^2^⁺ ions were placed into the predicted structures of AnGH92A and AnGH92B to evaluate their coordination geometries (Supplementary Fig. S4). The spatial arrangement of Ca^2^⁺-coordinating residues closely resembled that observed in the bacterial GH92 enzyme BT3990^30^.

GH92 *exo*-α-mannosidases typically catalyze hydrolysis via an inverting mechanism, in which a conserved glutamate functions as the general acid and an aspartate functions as the general base^30,37^. In the α-1,2-mannosidase BT3990, Glu533 and Asp644 serve these catalytic roles^30^. Structural superposition revealed that AnGH92A possesses Glu598 and Asp685 at the corresponding positions, whereas AnGH92B contains Glu588 and Asp675, indicating conservation of the catalytic acid–base pair across fungal and bacterial GH92 enzymes (Supplementary Figs. S3 and S4).

In bacterial GH92s, the Asp–Glu–Asp (DED) motif is frequently observed in the active site and is particularly characteristic of α-1,2-mannosidases^21^. By contrast, α-1,3-mannosidases exhibit greater variation, harboring Asn–Asp–Asp (NDD) or Asn–Glu–Asp (NED) motifs (Supplementary Fig. S3). Fungal GH92 enzymes exhibit even broader diversification. Among the *A. nidulans* proteins analyzed, AnGH92A, AnGH92B, and AnGH92E displayed the NDD motif, in which the third Asp is predicted to serve as the catalytic base. AnGH92C and AnGH92D, as well as GH92 enzymes from *Candida* species, instead contained an Asn–Ser–Asp (NSD) motif (Supplementary Fig. S3). Despite this diversity, the catalytically essential C-terminal Asp was conserved across all fungal and bacterial GH92 enzymes examined.

Sequence motifs associated with substrate specificity were also compared with those of bacterial GH92 enzymes. Bacterial α-1,2-mannosidases contain characteristic “His–Glu” and “Pro–Trp” motifs in their active sites, which are critical for recognizing α-1,2-linked mannose at the +1 subsite^30–32,36,42^. For example, in BT3990, His584–Glu585 and N-terminal Trp88 cooperatively secure the +1 mannose, with the His584–Glu585 pair forming part of the so-called 580-loop (residues 580–590)^30^. These motifs were absent in both AnGH92A and AnGH92B, and structural comparison confirmed that the 580-loop is also missing. This structural difference is likely one of the factors contributing to the lack of α-1,2-mannosidase activity in AnGH92A and AnGH92B (Fig. 3a; Supplementary Figs. S3 and S4).

### Conformational state of the −1 mannose residue in docking simulations

**Fig. 6:**
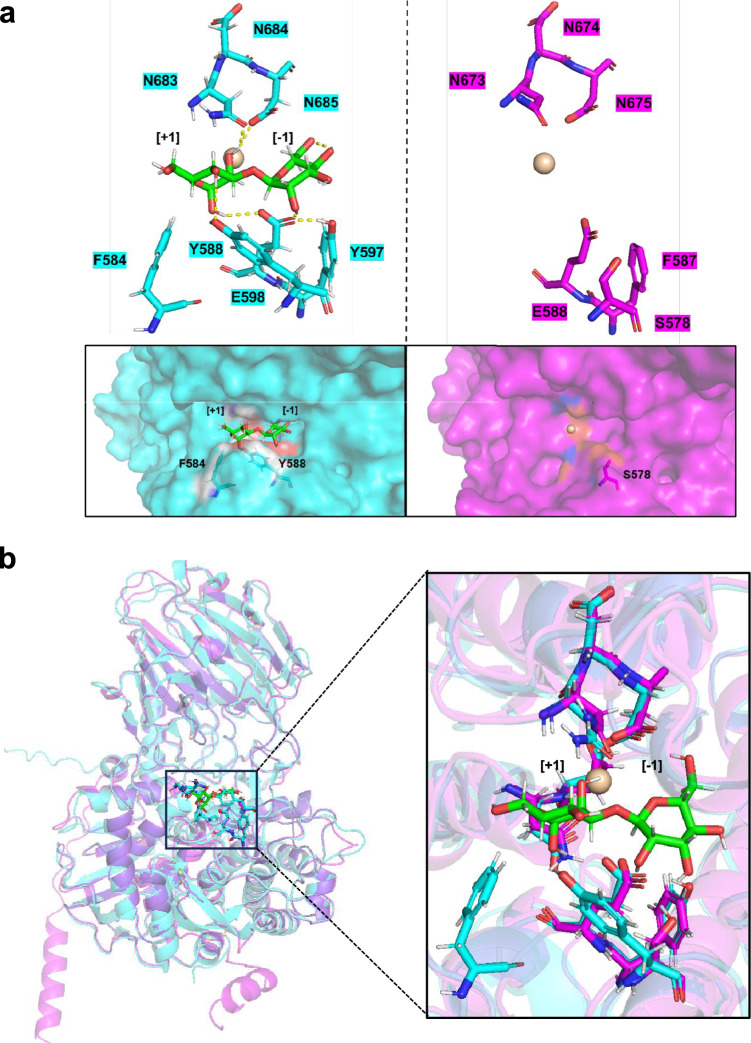
Structural comparison of AnGH92A and AnGH92B. (**a**) Enlarged views of AnGH92A (cyan) and AnGH92B (magenta). Structural models were generated using AlphaFold2. Docking simulations were performed using Ca^2^⁺-bound models of both enzymes. Substrate-binding sites of AnGH92A (cyan) and AnGH92B (magenta) docked with α-1,3-Man_2_ (green sticks) were predicted using MOE. Calcium ions are shown as spheres. Docking of α-1,3-Man_2_ to AnGH92B did not converge to a stable binding pose. (**b**) The AnGH92A–ligand complex model was superimposed onto the predicted structure of AnGH92B to compare active-site conformations. The right panel shows an enlarged view of the active-site region docked with α-1,3-Man_2_ (green sticks).

In GH92 family enzymes, a catalytic Ca^2^⁺ plays a crucial role in promoting substrate distortion during catalysis. Ca^2^⁺ often coordinates the O2 and O3 hydroxyl groups of the −1 mannose residue, inducing a transition of the pyranose ring from the relaxed chair (^4^*C*₁) conformation toward a distorted boat-like conformation (*E*₅/*B*₂,₅ → ^1^*S*₅) as part of the catalytic itinerary^30,31,37^. In this study, docking simulations were performed for both AnGH92A and AnGH92B, using α-1,3-Man_2_ (initially in its ^4^*C*₁ chair conformation) as the ligand, with a Ca^2^⁺ explicitly positioned in the active site to reproduce the catalytic geometry (Fig. 6). Docking converged only for AnGH92A; in contrast, α-1,3-Man_2_ did not adopt a stable binding pose in AnGH92B, and docking failed to converge on a reproducible enzyme–substrate complex (Fig. 6a). In the AnGH92A docking model, the −1 mannose residue remained in the chair conformation, with Ca^2^⁺ positioned closer to the +1 mannose residue rather than coordinating the O2 and O3 hydroxyl groups of the −1 subsite, and no significant distortion of the pyranose ring was observed. Because prior crystallographic and mechanistic studies have shown that conformational distortion occurs only during the catalytic process via Ca^2^⁺-assisted transition-state stabilization^30,37^, the docking result likely represents a Michaelis complex—a pre-reactive, ground-state binding mode prior to distortion toward the *E*₅/*B*₂,₅ or ^1^*S*₅ conformations characteristic of GH92 catalysis (Fig. 6a). To compare the active-site environments of the two enzymes, the AnGH92A–ligand complex model was superimposed onto the predicted structure of AnGH92B (Fig. 6b). This comparison highlights structural differences that likely underlie the failure of α-1,3-Man₂ to bind productively in AnGH92B.

### Structural features of fungal α-1,3-mannosidase

Although AnGH92A and AnGH92B share an overall similar fold, several notable structural differences were identified within their substrate-binding pockets. In AnGH92A, two aromatic residues—Phe584 and Tyr588—are positioned around the binding site (Fig. 6). By contrast, the corresponding positions in AnGH92B lack aromatic side chains; for example, the Tyr588 position in AnGH92A is occupied by Ser in AnGH92B. These differences arise from a short insertion sequence located near the FTSCYL motif, which is conserved only within the AnGH92A clade (Fig. 1; Supplementary Fig. S3). Surface comparisons of the binding pockets highlight clear isozyme-specific differences. In AnGH92A, Phe584 and Tyr588 partially overlay the substrate-binding cleft, creating a narrower and more hydrophobic environment that facilitates substrate positioning (Fig. 6a). Phe584 contributes to cleft narrowing, whereas Tyr588 is positioned to stabilize the bound substrate, likely through hydrogen bonding. Together, these residues enhance substrate affinity and stabilization, thereby contributing to the higher catalytic efficiency of AnGH92A. In contrast, the absence of these aromatic residues in AnGH92B results in a more open, solvent-exposed pocket that is less capable of supporting productive substrate binding (Fig. 6a). Consistent with these structural observations, only AnGH92A exhibited strong hydrolytic activity toward α-1,3-Man₂ in biochemical assays (Fig. 3a). Thus, the aromatic residue–rich loop formed by the FTSCYL-containing insertion represents a structural signature of the AnGH92A clade (Fig. 1a; Supplementary Fig. S3) and is likely a key determinant of α-1,3-mannosidase activity.

Comparative analysis with bacterial GH92 α-1,3-mannosidases such as BT3130 and B035DRAFT_03340^GH92^ showed that the catalytic and Ca^2^⁺-coordinating residues required for activity are well conserved^31,33^. However, substantial differences were observed in residues shaping the substrate-binding pocket (Supplementary Figs. S3 and S5a–c). At the −1 subsite, Gly100 and Asp381 of AnGH92A correspond to Gly92 and Asp353 in BT3130, whereas Met400 of BT3130 is substituted by Gln428 in AnGH92A. Phe219 of AnGH92A also occupies a position analogous to Trp198 and Trp390 in BT3130. At the +1 subsite, bacterial enzymes employ multiple aromatic residues (e.g., tryptophans), whereas AnGH92A lacks these canonical aromatic stacking elements (Supplementary Figs. S3 and S5a–c). Instead, a short insertion containing the FTSCYL motif contributes aromatic residues Phe584, Tyr588, and Tyr597 to the pocket, creating a distinct fungal-specific aromatic environment around the binding site (Supplementary Figs. S3 and S5a–c). By contrast, even among bacterial GH92 α-1,3-mannosidases, pocket architectures vary; in B035DRAFT_03340^GH92^, the tryptophan residues at the +1 subsite sit farther from the substrate, creating a broader and more flexible pocket (Supplementary Fig. S5d–f). These comparisons reveal that GH92 α-1,3-mannosidases exhibit considerable diversity in their substrate-binding clefts—from tightly enclosed to widely open configurations—largely shaped by the presence, absence, or positioning of aromatic residues.

### Site-directed mutagenesis reveals catalytic residues in AnGH92A

To identify residues essential for catalysis in AnGH92A, site-directed mutagenesis was performed (Supplementary Fig. S6). Two aromatic residues located within the substrate-binding pocket—Phe584 and Tyr588—were substituted with Ala and Ser, respectively (F584A, Y588S). The Y588S substitution was designed to mimic the corresponding Ser residue in AnGH92B. Putative catalytic residues Glu598, Asp684, and Asp685 were replaced with either alanine (E598A, D684A, D685A) or charge-neutralizing variants (E598Q, D684N). An additional mutant, N683A, was generated because the corresponding residue has been implicated in GH92 catalysis^30^.

Enzyme assays using 4NP-Man showed that all variants except F584A exhibited a >90% reduction in activity relative to the wild-type enzyme (Table S3). Substitutions at Glu598 and Asp685 resulted in complete loss of hydrolytic activity, while Y588S, N683A, D684N, and D684A retained only minimal residual activity. By contrast, F584A maintained moderate activity (Table S3). These data indicate that Glu598 and Asp685 function as the catalytic acid and catalytic base, respectively, consistent with the conserved catalytic machinery of GH92 enzymes. Hydrolysis assays using α-manno-oligosaccharides containing α-1,3-linkages (Man₂, Man₃, Man₄, Man₅) and yeast α-mannan supported these conclusions (Supplementary Fig. S7). TLC analyses showed that the F584A mutant was able to produce mannose, albeit at reduced levels compared with the wild type, whereas Y588S produced only trace hydrolysis products. Mutants E598Q/A and D685A were completely inactive (Supplementary Fig. S7).

Collectively, these results demonstrate that in AnGH92A, Glu598 and Asp 685 act as the catalytic acid and catalytic base residues, respectively, consistent with the catalytic architecture reported for other GH92 enzymes. In addition, the mutagenesis analysis clarifies the functional roles of aromatic residues within the substrate-binding pocket. Although the F584A variant retained partial activity, the Y588S variant showed a pronounced reductions in activity. These findings indicate that Phe584 and Tyr588 contribute to substrate binding and stabilization, with Tyr588 being particularly essential for productive catalysis in AnGH92A.

## Discussion

In this study, we demonstrated that AnGH92A from the filamentous fungus *A. nidulans* functions as an α-1,3-mannosidase. To our knowledge, this represents the first functional characterization of a eukaryotic GH92 enzyme and provides new insight into a glycoside hydrolase family that has been specifically retained in fungi.

Structural modeling and site-directed mutagenesis analyses identified Glu598 and Asp685 as the catalytic acid and catalytic base residues, respectively (Supplementary Fig. S7; Table S3). In addition, aromatic residues such as Tyr588 and Phe584, located within the substrate-binding pocket, were found to be essential for substrate recognition and contribute to differences in substrate affinity and catalytic efficiency (Fig. 6). These findings are consistent with observations in other glycoside hydrolases, where aromatic residues frequently participate in substrate stabilization. For example, substitution of a key Tyr residue with Ser in *O*-GlcNAcase, abolishes substrate interactions and leads to a marked decrease in activity^43^. Likewise, our structural analysis of AnGH92A revealed that the arrangement of aromatic residues surrounding the +1 mannose ring is essential for catalysis. The inactivation observed in the Y588S mutant further underscores the essential role of aromatic residues in substrate recognition and fixation. Collectively, these findings indicate that fungal GH92 enzymes have diverged from their bacterial counterparts, acquiring specialized structural features for substrate recognition.

In contrast to AnGH92A, the physiological substrate of AnGH92B remains unclear. Although AnGH92B shares 46.2% sequence identity with AnGH92A, it displayed no detectable hydrolytic activity toward manno-oligosaccharides. Nevertheless, AnGH92B hydrolyzed the artificial substrate 4NP-Man, indicating that it can cleave mannosidic linkages. Structural modeling revealed that the substrate-binding pocket of AnGH92B is broader and more solvent-exposed than that of AnGH92A. The higher *K*_m_ value observed for AnGH92B (Table 1) may reflect its lower affinity for hydrophilic substrates, consistent with a more open pocket architecture. These features suggest that AnGH92B may preferentially act on bulkier or hydrophobic mannosides, such as lipid-linked glycoconjugates^44^, rather than soluble manno-oligosaccharides. Further biochemical analyses will be required to identify its physiological substrate.

An intriguing aspect of GH92 biology is their uneven distribution among fungi. GH92 enzymes are absent in budding yeasts such as *S. cerevisiae* yet are retained in pathogenic fungi such as *C. albicans*, which form pseudohyphae^21^. This taxonomic pattern suggests that GH92 enzymes may function in fungal developmental processes, including hyphal elongation and differentiation, in addition to nutrient acquisition. *A. nidulans* encodes a diverse repertoire of glycoside hydrolases that support the utilization of various polysaccharides as carbon sources^45^. Given that AnGH92A carries a predicted signal peptide, it was initially presumed to be secreted extracellularly to degrade α-mannan. However, *A. nidulans* failed to grow when yeast α-mannan or α-manno-oligosaccharides were provided as their sole carbon source (data not shown), indicating that fungal GH92 enzymes may not primarily function in external α-mannan catabolism. Instead, several lines of evidence point to a developmental role. Expression of the *A. niger* ortholog of AnGH92A (accession number: CAK40038.1) is strongly induced under carbon starvation and significantly reduced in *flbA*-deficient strains lacking FlbA, a key regulator of conidiation^46–49^. Because *ΔflbA* mutants are unable to form conidia, these observations suggest that AnGH92A may contribute to aerial hyphal development and conidiation, particularly under nutrient-limited conditions. Future *in vivo* studies will be essential to delineate the specific physiological contexts in which fungal GH92 enzymes operate.

Beyond its biological significance, AnGH92A also has potential industrial applications. Its ability to cleave α-1,3-linkages enables the controlled removal of α-manno-oligosaccharide side chains. α-Manno-oligosaccharides are well-established prebiotics used in aquaculture and livestock production to promote intestinal health and modulate immune responses^50–52^. The oligosaccharides produced by removing α-1,3-linked mannose residues may therefore exhibit unique functional properties, offering opportunities for expanding prebiotic formulations and developing new functional feed ingredients.

In conclusion, this work presents the first functional characterization of a eukaryotic GH92 enzyme and establishes that AnGH92A acts as an α-1,3-mannosidase with weak activity toward α-1,4-linkages. These findings provide foundational insight into the catalytic architecture, substrate specificity, and evolutionary diversification of fungal GH92 enzymes. Taken together, our results advance the understanding of fungal-specific carbohydrate-active enzymes and lay the groundwork for future studies exploring their roles in fungal development, cell wall remodeling, and potential applications in biotechnology and prebiotic innovation.

## Supplementary Information

Supplementary Information.

## Data Availability

All data generated or analyzed in this study are included in this published article and its supplementary information files.
