# Supplementary material for: Biochemical characterization of the α-1,3-mannosidase AnGH92A from Aspergillus nidulans
Source: Sci Rep. 2026 Jan 5;16:4297. doi: 10.1038/s41598-025-34466-6 (PMC12858847; doi:10.1038/s41598-025-34466-6)
Supplement: Supplementary file 1 — Supplementary Information. [file 41598_2025_34466_MOESM1_ESM.docx]

**Supplementary information**

**Biochemical characterization of the α-1,3-mannosidase AnGH92A from *Aspergillus nidulans***

Ryutaro Nishigaki^1^, Hiromitsu Suzuki^1^, Ryohei Tsukada^1^, Ken Miyazawa^2^, Masashi Kato^1^, and Motoyuki Shimizu^1^*****

^1^ Faculty of Agriculture, Meijo University, Nagoya, Japan

^2^ Department of Fungal Infection, National Institute of Infectious Diseases, Japan Institute for Health Security, Tokyo, Japan

*Corresponding author

Motoyuki Shimizu

Faculty of Agriculture, Meijo University,

Nagoya, Japan

[moshimi@meijo-u.ac.jp](mailto:moshimi@meijo-u.ac.jp)

(ORCID; 0000-0002-6907-6367)

**
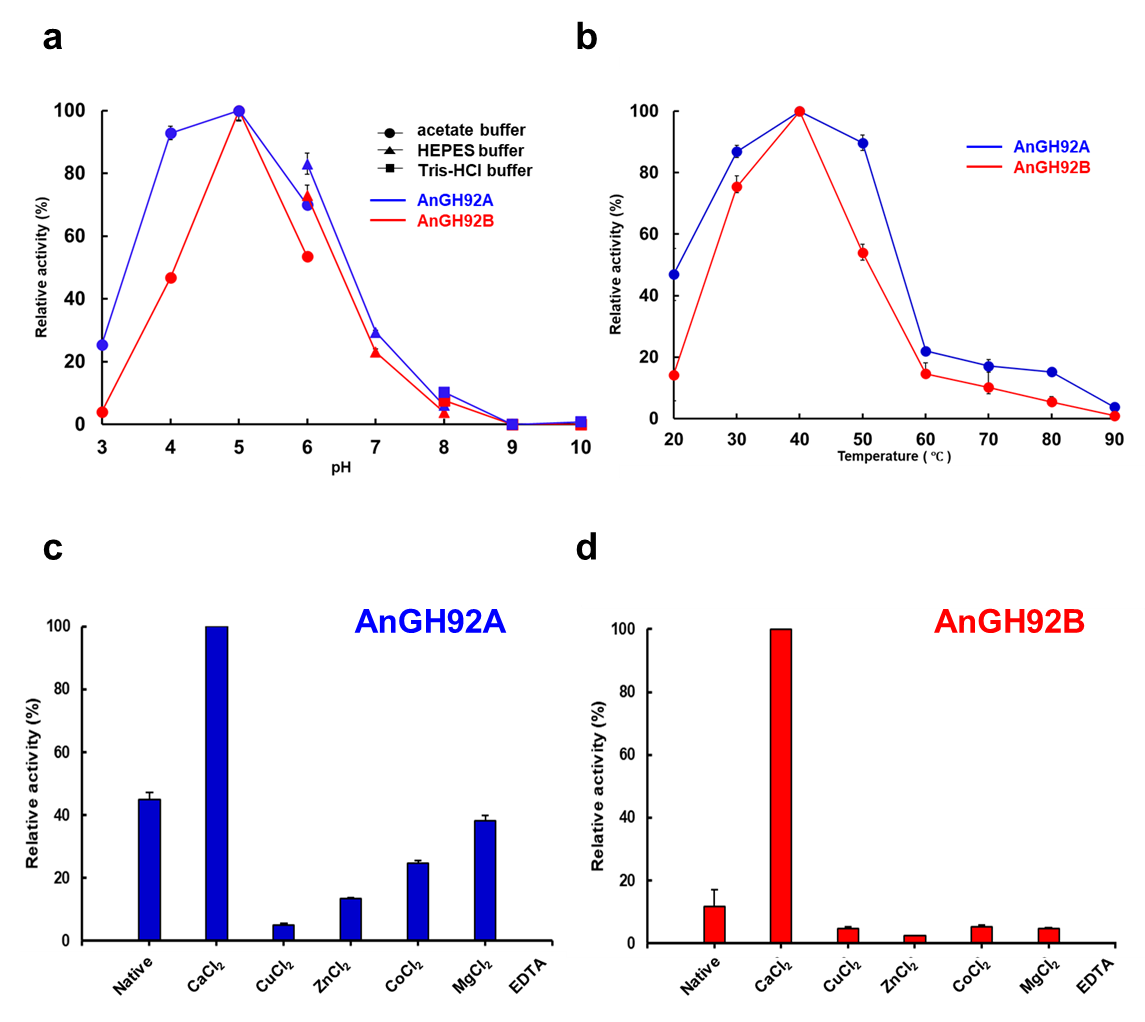
**

**Fig. S1. Optimal conditions for AnGH92A and AnGH92B.**

**(a)** Optimal pH profiles of AnGH92A (blue) and AnGH92B (red). Enzyme reactions were performed across pH 3.0–10.0 using 50 mM sodium acetate (pH 3.0–6.0; ●), 50 mM HEPES-NaOH (pH 6.0–8.0; ▲), and 50 mM Tris-HCl (pH 8.0–10.0; ■).

**(b)** Optimal temperature profiles of AnGH92A (blue) and AnGH92B (red), assessed at 20–90 °C.

**(c, d)** Effects of metal ions on AnGH92A (c) and AnGH92B (d). Enzymes were preincubated with the indicated additives at 4 °C for 24 h, and residual activity was measured at pH 5.0 and 37 °C for 30 min. Activity in the absence of additives was set to 100%. All activities were assayed using 4NP-Man as the substrate. Values represent the mean ± standard error of three independent experiments.


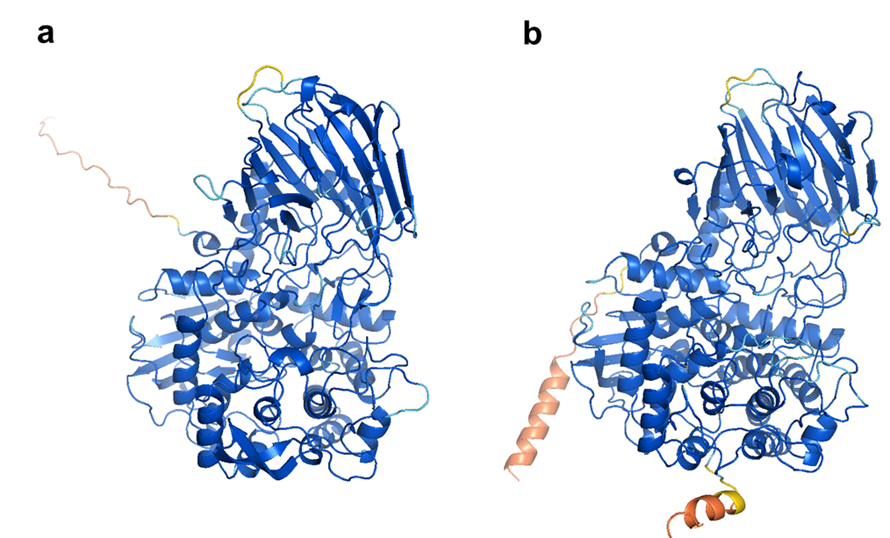


**Fig. S2. Structural comparison of AnGH92A and AnGH92B.**

Predicted structural models of **(a)** AnGH92A and **(b)** AnGH92B generated using AlphaFold2. Structural confidence is indicated by pLDDT scores: navy blue (<100), cyan (<90), yellow (<70), orange (<50).

**Fig. S3. Amino acid sequence alignment of GH92 enzymes.**

Multiple sequence alignment of GH92 enzymes from *Aspergillus nidulans* (AnGH92A–E), along with AnGH92A-clade orthologs from *Penicillium digitatum* (QQK46283.1), *Aspergillus oryzae* (BAE65809.1), and *Aspergillus niger* (CAK40038.1), as well as three *Candida* species: *C. albicans* SC5314 (AOW31314.1), *C. dubliniensis* CD36 (CAX40133.1), and *C. parapsilosis* CDC317 (CCE42865.1). Representative bacterial GH92 enzymes were also included: α-1,3-mannosidases (BT3130, BT3994, NixK, HMPREF1534_02484, BT3773, Sca2/3Man2693), α-1,2-mannosidases (BT3990, EfMan-I, NnGH92, BT3962, BT3784), and α-1,4-mannosidases (BT4073, BT3965). Alignments were generated using ClustalW. Conserved residues and characteristic motifs are highlighted as follows: catalytic residues (magenta), Ca²⁺-binding residues (yellow), α-1,2-mannosidase-conserved motifs (green), GH92-common residues in bacteria (pink), GH92-common residues in fungi (blue), residues conserved across all GH92 enzymes (gray), residues shared among α-1,3-mannosidases (purple), bacterial α-1,3-mannosidase-specific residues (orange), and the unique motif of AnGH92A (cyan).


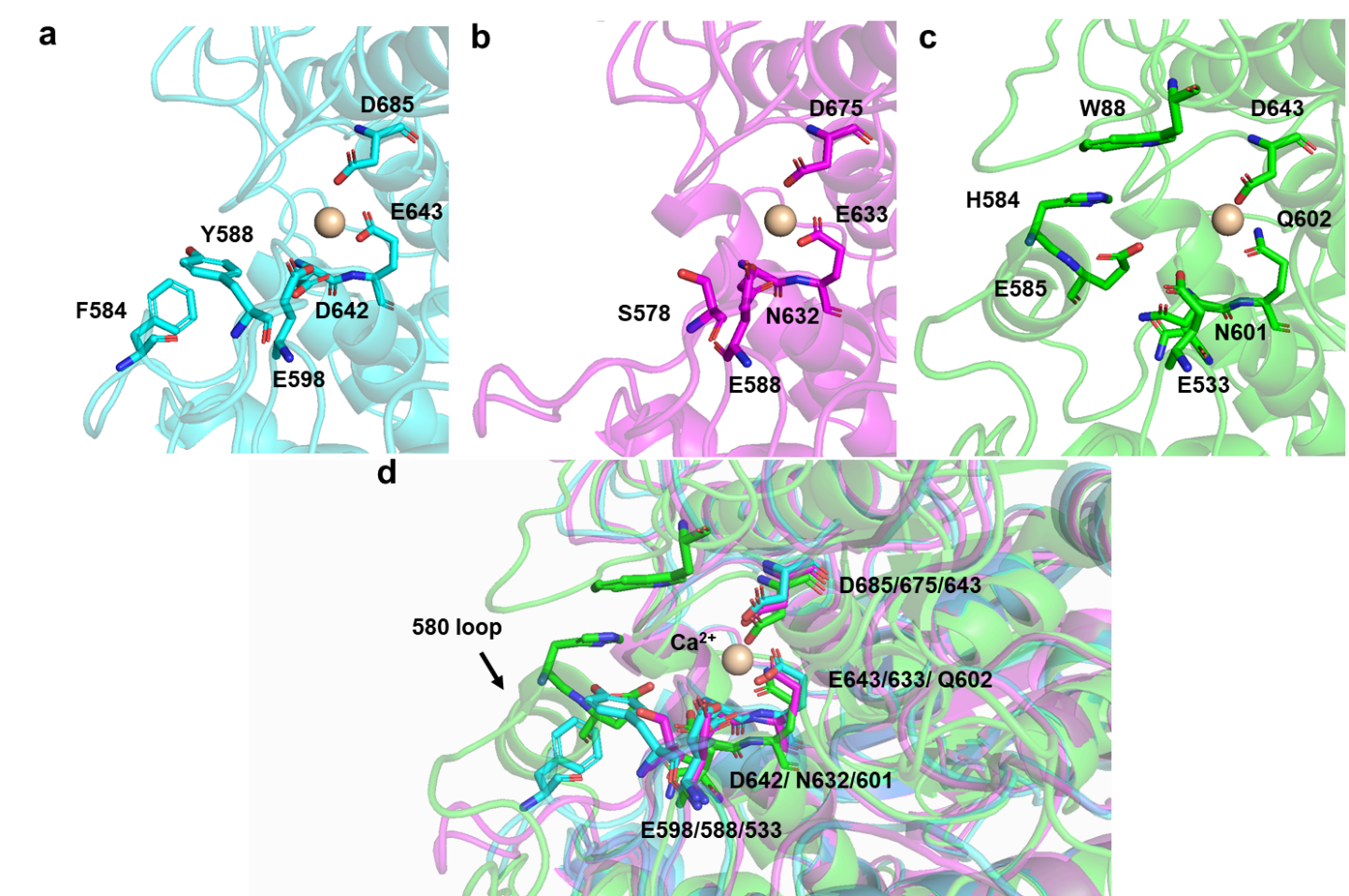


**Fig. S4. Structural comparison of AnGH92A, AnGH92B, and bacterial α-1,2-mannosidase BT3990**

Enlarged views (a–c) and superimposed structures (d) of AnGH92A (cyan), AnGH92B (magenta), and the bacterial α-1,2-mannosidase BT3990 (green; PDB ID: 2WW1). Structural models of AnGH92A and AnGH92B were generated using AlphaFold2. Ca²⁺ was positioned based on the coordinates of the BT3990 crystal structure (PDB ID: 2WW1). Conserved Ca²⁺-binding residues and catalytic residues are indicated. Structural differences in the loop regions surrounding the predicted +1 subsite are highlighted to illustrate structural variations among these GH92 enzymes.


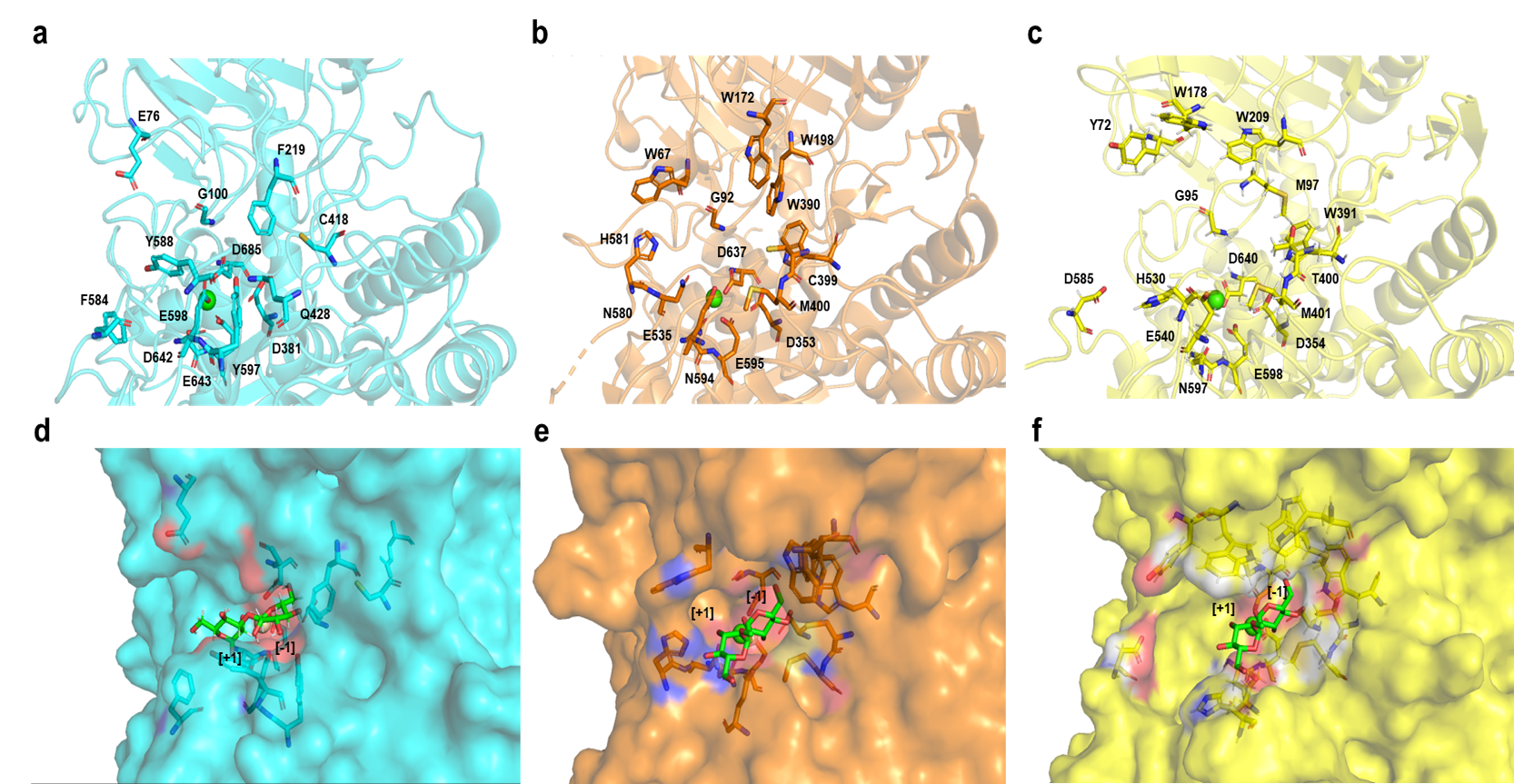


**Fig. S5. Structural comparison of AnGH92A and bacterial α-1,3-mannnosidases**

Enlarged views of AnGH92A (cyan) compared with the bacterial α-1,3-mannosidase BT3130 (orange; PDB ID: 6F8Z) and B035DRAFT_03340^GH92^ (yellow; PDB ID: 7ZGM) ligand-free (a–c) and ligand-bound (d–f) states. The structural model of AnGH92A was generated using AlphaFold2. A Ca²⁺ ion was positioned based on the coordinates of the BT3990 crystal structure (PDB ID: 2WW1), and docking simulations were performed using the Ca²⁺-bound AnGH92A model.


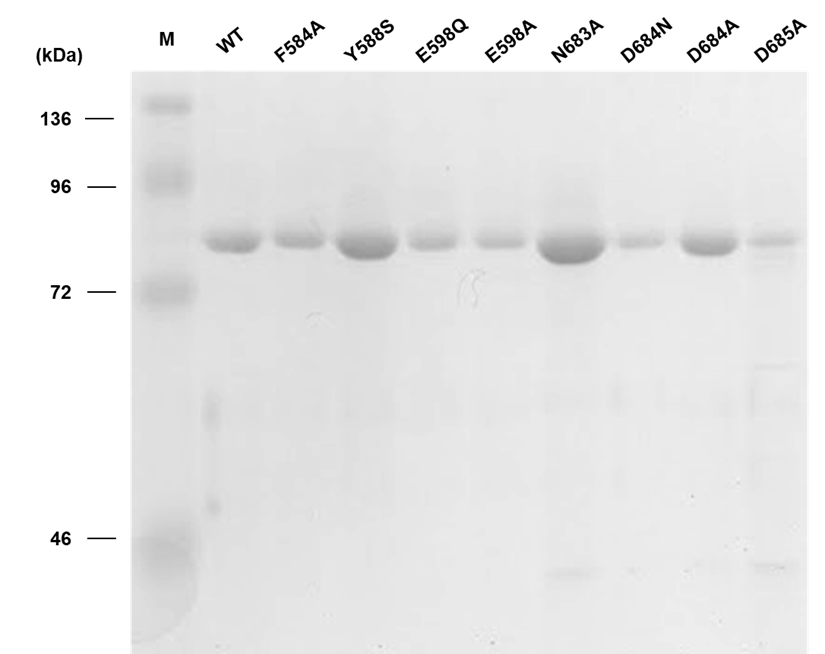


**Fig. S6. SDS–PAGE analysis of purified AnGH92A variants.**

Lane M, protein molecular mass markers; purified wild-type AnGH92A (WT) and variants F584A, Y588S, E598Q, E598A, N683A, D684N, D684A, and D685A.


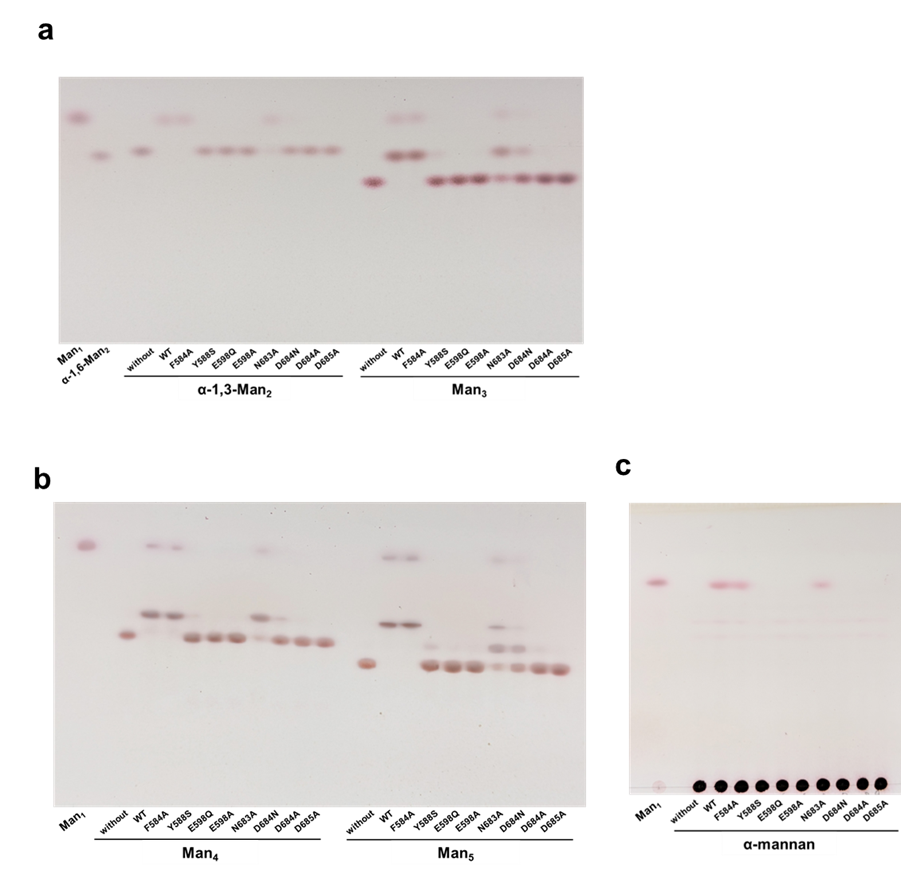


**Fig. S7. TLC analysis of reaction products from α-manno-oligosaccharides and α-mannan hydrolyzed by AnGH92A variants.**

**(a, b)** Reaction products generated from α-manno-oligosaccharides; **(c)** reaction products generated from α-mannan. Reactions were performed at 40 °C and pH 5.0 for 30 min. Mannose (Man₁) is indicated. Substrates: α-1,2-mannobiose (α-1,2-Man₂); α-1,3-mannobiose (α-1,3-Man₂); α-1,4-mannobiose (α-1,4-Man₂); α-1,6-mannobiose (α-1,6-Man₂); *O*-α-d-mannopyranosyl-(1→3)-*O*-[α-d-mannopyranosyl-(1→6)]-α-d-mannopyranose (Man₃); *O*-α-d-mannopyranosyl-(1→3)-*O*-[α-d-mannopyranosyl-(1→6)]-*O*-α-d-mannopyranosyl-(1→6)-d-mannose (Man₄); *O*-α-d-mannopyranosyl-(1→3)-*O*-[α-d-mannopyranosyl-(1→6)]-*O*-α-d-mannopyranosyl-(1→6)-*O*-[α-d-mannopyranosyl-(1→3)]-d-mannose (Man₅). “Without” indicates reactions performed in the absence of enzymes.

**Table S1. Primers used in this study.**

| **Primer name** | **Gene ID** | **Primer sequence (5′→3′)** |
| --- | --- | --- |
| **Primers used to clone GH92 genes** | | |
| AnGH92A_Fw | ANIA_10672 | AGAGAGGCTGAAGCTGGGCGAAAACTCCGCAGCGA |
| AnGH92A_Rv |  | CGGCCGGCTGGGCCACGTGTTACGCATATTCCGAC |
| AnGH92B_Fw | ANIA_01197 | AGAGAGGCTGAAGCTGGGAGCGGTCTCAGCGCC |
| AnGH92B_Rv |  | CGGCCGGCTGGGCCACGTGTCACAACACATCCGAA |
| AnGH92C_Fw | ANIA_03054 | AGAGAGGCTGAAGCTGGGGCAGTGGATTATTCTC |
| AnGH92C_Rv |  | CGGCCGGCTGGGCCACGTGTCAGACTTCGACATGT |
| **Primers used for site-directed mutagenesis (AnGH92A variants)** | | |
| AnGH92A_F584A_Fw | ANIA_10672 | AGCCCGCTATTGAACGCCACGTCCTGCTATCTG |
| AnGH92A_F584A_Rv |  | CAGATAGCAGGACGTGGCGTTCAATAGCGGGCT |
| AnGH92A_Y588S_Fw |  | AACTTCACGTCCTGCAGTCTGAATGCCGACGGG |
| AnGH92A_Y588S_Rv |  | CCCGTCGGCATTCAGACTGCAGGACGTGAAGTT |
| AnGH92A_E598Q_Fw |  | GGGCACGAGACTTATCAGGGTAGTTGCTGGCTT |
| AnGH92A_E598Q_Rv |  | AAGCCAGCAACTACCCTGATAAGTCTCGTGCCC |
| AnGH92A_E598A_Fw |  | GGGCACGAGACTTATGCGGGTAGTTGCTGGCTT |
| AnGH92A_E598A_Rv |  | AAGCCAGCAACTACCCGCATAAGTCTCGTGCCC |
| AnGH92A_N683A_Fw |  | TCTGGCATCCCCGGTGCCGACGACAGCGGCGCA |
| AnGH92A_N683A_Rv |  | TGCGCCGCTGTCGTCGGCACCGGGGATGCCAGA |
| AnGH92A_D684N_Fw |  | GGCATCCCCGGTAACAACGACAGCGGCGCAATG |
| AnGH92A_D684N_Rv |  | CATTGCGCCGCTGTCGTTGTTACCGGGGATGCC |
| AnGH92A_D684A_Fw |  | GGCATCCCCGGTAACGCCGACAGCGGCGCAATG |
| AnGH92A_D684A_Rv |  | CATTGCGCCGCTGTCGGCGTTACCGGGGATGCC |
| AnGH92A_D685A_Fw |  | ATCCCCGGTAACGACGCCAGCGGCGCAATGGGC |
| AnGH92A_D685A_Rv |  | GCCCATTGCGCCGCTGGCGTCGTTACCGGGGAT |

Primers used for cloning and site-directed mutagenesis of GH92 genes. Gene-specific primers were designed based on the *Aspergillus nidulans* genomic sequence available from the Joint Genome Institute (<https://mycocosm.jgi.doe.gov/Aspnid1/Aspnid1.home.html>).

**Table S2. Accession numbers and organisms of GH92 sequences used for phylogenetic analysis in Fig. 1.**

| **Accession No.** | **Annotation** | **Organism** |
| --- | --- | --- |
| AAO79095.1 | mannosyl-oligosaccharide α-1,2-mannosidase | *Bacteroides thetaiotaomicron* VPI-5482 |
| AAO77306.1 | mannosyl-oligosaccharide α-1,2-mannosidase | *Bacteroides thetaiotaomicron* VPI-5482 |
| AAO78633.1 | α-mannosidase | *Bacteroides thetaiotaomicron* VPI-5482 |
| AAO79067.1 | α-1,2-mannosidase | *Bacteroides thetaiotaomicron* VPI-5482 |
| AAO78889.1 | α-1,2-mannosidase | *Bacteroides thetaiotaomicron* VPI-5482 |
| AAO77736.1 | α-mannan α-1,2-mannosidase | *Bacteroides thetaiotaomicron* VPI-5482 |
| AAO79197.1 | α-1,2-mannosidase | *Bacteroides thetaiotaomicron* VPI-5482 |
| AAO76985.1 | α-1,3-mannosidase | *Bacteroides thetaiotaomicron* VPI-5482 |
| AAO78236.1 | α-1,3-mannosidase | *Bacteroides thetaiotaomicron* VPI-5482 |
| AAO79099.1 | mannosyl-oligosaccharide α-1,3-mannosidase | *Bacteroides thetaiotaomicron* VPI-5482 |
| AAO79068.1 | α-mannosidase | *Bacteroides thetaiotaomicron* VPI-5482 |
| AAO78878.1 | α-1,3-mannosidase | *Bacteroides thetaiotaomicron* VPI-5482 |
| AAO79096.1 | mannosyl-oligosaccharide α-1,3-mannosidase | *Bacteroides thetaiotaomicron* VPI-5482 |
| AAO76876.1 | mannosyl-oligosaccharide α-1,3-mannosidase | *Bacteroides thetaiotaomicron* VPI-5482 |
| AAO76139.1 | α-mannosidase | *Bacteroides thetaiotaomicron* VPI-5482 |
| AAO79198.1 | α-1,4-mannosidase | *Bacteroides thetaiotaomicron* VPI-5482 |
| AAO78963.1 | mannosyl-oligosaccharide α-1,3-mannosidase | *Bacteroides thetaiotaomicron* VPI-5482 |
| AAO79178.1 | α-1,4-mannosidase | *Bacteroides thetaiotaomicron* VPI-5482 |
| AAO79070.1 | α-1,4-mannosidase | *Bacteroides thetaiotaomicron* VPI-5482 |
| AAO77218.1 | α-mannosidase | *Bacteroides thetaiotaomicron* VPI-5482 |
| AAO78636.1 | α-1,4-mannosidase | *Bacteroides thetaiotaomicron* VPI-5482 |
| AFK83605.1 | [*N*-glycan] (Man-1-Pi-6-Man-specific) uncapping α-mannosidase | *Cellulosimicrobium cellulans* |
| AFK83606.1 | α-mannosidase | *Cellulosimicrobium cellulans* |
| AFK83608.1 | mannosyl-oligosaccharide α-1,2-mannosidase | *Cellulosimicrobium cellulans* |
| EOK07369.1 | exo-α-1,2-mannosidase | *Enterococcus faecalis* ATCC 10100 |
| AAO81948.1 | [*N*-glycan] exo-α-1,2-mannosidase | *Enterococcus faecalis* V583 |
| BAA76709.1 | α-1,2-mannosidase / *exo*-α-1,2-mannosidase | *Microbacterium sp.* M-90 |
| CAD7619346.1 | *exo*-α-1,2-mannosidase | *Neobacillus novalis* |
| WP_005941513.1 | mannosyl-oligosaccharide α-1,3-mannosidase | *Phocaeicola massiliensis* |
| AFD08953.1 | mannosyl-oligosaccharide α-1,2/1,3-mannosidase | *Solitalea canadensis* DSM 3403 |
| AAK76202.1 | mannosyl-oligosaccharide α-1,2-mannosidase | *Streptococcus pneumoniae* TIGR4 |
| AAM42166.1 | [*N*-glycan] α-1,3-mannosidase | *Xanthomonas campestris pv. campestris str.* ATCC 33913 |
| QGN17649.1 | hypothetical protein FIM1_4856 | *Kluyveromyces marxianus* |
| CCE42865.1 | hypothetical protein CPAR2_205080 | *Candida parapsilosis* |
| CAX40133.1 | α-1,2-mannosidase, putative | *Candida dubliniensis* CD36 |
| AOW31314.1 | hypothetical protein CAALFM_CR06030CA | *Candida albicans* SC5314 |
| QMW47886.1 | hypothetical protein G4B11_011404 | *Aspergillus flavus* |
| QMW48457.1 | hypothetical protein G4B11_011975 | *Aspergillus flavus* |
| QMW46420.1 | hypothetical protein G4B11_009875 | *Aspergillus flavus* |
| QMW44975.1 | hypothetical protein G4B11_008395 | *Aspergillus flavus* |
| QMW38730.1 | hypothetical protein G4B11_001966 | *Aspergillus flavus* |
| QMW37742.1 | hypothetical protein G4B11_000978 | *Aspergillus flavus* |
| BCS07710.1 | hypothetical protein ALUC_20080A | *Aspergillus luchuensis* |
| BCS09855.1 | hypothetical protein ALUC_30672A | *Aspergillus luchuensis* |
| BCS11709.1 | hypothetical protein ALUC_41049A | *Aspergillus luchuensis* |
| BCS15002.1 | hypothetical protein ALUC_70235A | *Aspergillus luchuensis* |
| BCS13071.1 | hypothetical protein ALUC_51117S | *Aspergillus luchuensis* |
| CAK45343.1 | unnamed protein product | *Aspergillus niger* |
| CAK40038.1 | unnamed protein product | *Aspergillus niger* |
| CAK41526.1 | unnamed protein product | *Aspergillus niger* |
| CAK48516.1 | unnamed protein product | *Aspergillus niger* |
| CAK48600.1 | unnamed protein product | *Aspergillus niger* |
| BAE55274.1 | unnamed protein product | *Aspergillus oryzae* RIB40 |
| BAE62692.1 | unnamed protein product | *Aspergillus oryzae* RIB40 |
| BAE64139.1 | unnamed protein product | *Aspergillus oryzae* RIB40 |
| BAE65809.1 | unnamed protein product | *Aspergillus oryzae* RIB40 |
| BAE66337.1 | unnamed protein product | *Aspergillus oryzae* RIB40 |
| XP_050468271.1 | α-1,2-mannosidase family protein, putative (AnGH92A) | *Aspergillus nidulans* FGSC A4 |
| XP_050469023.1 | α-1,2-mannosidase family protein, putative (AnGH92B) | *Aspergillus nidulans* FGSC A4 |
| XP_050468469.1 | α-1,2-mannosidase family protein, putative (AnGH92C) | *Aspergillus nidulans* FGSC A4 |
| XP_659929.1 | α-1,2-mannosidase family protein, putative (AnGH92D) | *Aspergillus nidulans* FGSC A4 |
| XP_661368.1 | α-1,2-mannosidase family protein, putative (AnGH92E) | *Aspergillus nidulans* FGSC A4 |
| QQK43990.1 | α-1,2-mannosidase, putative | *Penicillium digitatum* |
| QQK46811.1 | α-1,2-mannosidase family protein, putative | *Penicillium digitatum* |
| QQK46283.1 | putative α-1,2-mannosidase | *Penicillium digitatum* |
| EAA31188.1 | α-1,2-mannosidase subfamily | *Neurospora crassa* OR74A |
| WZH40817.1 | glycosyl hydrolase | *Fusarium acuminatum* |
| CEI67665.1 | unnamed protein product | *Fusarium venenatum* |
| UKZ85342.1 | hypothetical protein TrAFT101_001206 | *Trichoderma asperellum* |
| UKZ85555.1 | hypothetical protein TrAFT101_001410 | *Trichoderma asperellum* |
| UKZ87297.1 | hypothetical protein TrAFT101_003105 | *Trichoderma asperellum* |
| UKZ94676.1 | hypothetical protein TrAFT101_009528 | *Trichoderma asperellum* |
| UKZ86424.1 | hypothetical protein TrAFT101_002256 | *Trichoderma asperellum* |

**Table S3. Specific activities of wild-type and variant AnGH92A enzymes.**

| **Enzyme** | **Activity (µmol·min⁻¹·mg⁻¹)** |
| --- | --- |
| WT | 15 ± 4.6 |
| F584A | 8.9 ± 1.8 |
| Y588S | 0.46 ± 0.19 |
| E598Q | NA* |
| E598A | NA |
| N683A | 0.98 ± 0.19 |
| D684N | 1.4 × 10^-3^ ± 4.5 × 10^-5^ |
| D684A | 1.1 × 10^-3^ ± 4.5 × 10^-5^ |
| D685A | NA |

α-Mannosidase activities of the AnGH92A wild type and its variants (F584A, Y588S, E598Q, E598A, N683A, D684N, D684A, and D685A) were measured using 4-nitrophenyl α-d-mannopyranoside (4NP-Man) as the substrate. Activities are expressed as specific activity (µmol·min⁻¹·mg⁻¹ protein). Values represent the mean ± standard error from three independent experiments.

*NA: No detectable activity.
